# The Secret Lives of Pluripotent Cells: There and Back Again

**DOI:** 10.3390/genes1010004

**Published:** 2010-03-09

**Authors:** Paolo Cinelli

Embryonic stem cells (ESCs) and induced pluripotent stem cells (IPSCs) hold great promise for the therapeutic treatment of human diseases, but their functional similarity, their stability and especially the mechanism underlying their derivation are not yet clearly explained. 

Embryonic development in mammalians is a very exciting process that starts with the generation of a totipotent unicellular zygote, continues with a series of precisely coordinated cellular differentiation processes, in which the newly generated cells undergo a progressive restriction of developmental potential, and is finally fully accomplished with the generation of an organism composed of more than 200 different highly specialized unipotent cell types. This extremely complex process is coordinated not only through tissue-specific transcriptional regulatory proteins, but also by modifications of higher-order chromatin structures accompanied by changes of the chromatin organization at the level of individual genes.

Nevertheless, subsets of self-renewing multipotent and unipotent cells, the so-called stem cells, are set aside at various steps of development and have been identified and described in several mammalian tissues. They are characterized by their ability to self-renew and to differentiate into a diverse range of specialized cell types: act as a repair system for the body, replenish specialized cells, and maintain the normal turnover of regenerative organs, such as blood, skin, or intestinal tissues.

Embryonic stem cells (ESCs) are a very particular group of pluripotent stem cells, which can be isolated from the inner cell mass of blastocyst stage embryos and possess the capacity to contribute to the formation of all cells of an adult organism. In reality, the fact that stable and self-renewing pluripotent ESC lines can be derived from pre-implantation embryos is very interesting, because, *in vivo*, pluripotent cells in early embryos proliferate only for a very short time and become cells with a more restricted developmental potential; ESCs are therefore considered a cell culture artifact. Their main characteristic *in vitro* is that they can self-renew indefinitely while maintaining their pluripotent state, a condition that is sustained through a specific transcriptional hierarchy that controls the process of self-renewal. A very fascinating scientific challenge is the elucidation of how cells with such capacities are established and propagated.

ESCs are not homogeneous, every individual cell exhibits variable expression levels of pluripotency factors, for example Nanog, and also have distinct probabilities of self-renewal, which leads to the assumption that they may also have differing developmental potential. Their capacity to be able to generate all cells from the body makes them a valuable tool for future regenerative medicine applications. 

The recent discovery that differentiated cells can be reprogrammed to pluripotency by transduction with four genes important for pluripotency, the so-called Yamanaka factors (Sox2, Oct4, Klf4 and c-Myc), not only widens our understanding of the concept of pluripotency but also opens new ways to the efficient derivation of patient-specific, autologous stem cells, which possess considerable potential for the study and for the treatment of human diseases. Using mouse models, it was recently shown that skin cells reprogrammed with the Yamanaka factors can be used to treat the symptoms of Parkinson’s disease [1] and sickle cell anemia [2]. Nonetheless, although the discovery of iPSCs is an incredible step forward in stem-cell research, at the moment this technology represents only the beginning of a long road. The protocols used for the generation of iPSCs are still linked to the use of viral vectors and oncogenes, which makes these cells currently more of a great research application than a therapeutic tool. Therefore, the necessity of creating these cells without permanent genetic modifications will determine in the near future the research in this field. 

However, before the real therapeutic potential of iPSCs can be fully exploited, it will be necessary to clearly understand the processes that maintain pluripotency in these cells and signal differentiation. It will also be necessary to clarify if iPSCs really have the same properties and potentials as embryonic stem cells, because they might differ in their abilities to differentiate in the same way that embryonic stem cells seem to. 

Furthermore, more extensive studies directed towards the identification of the best cell types for reprogramming are still necessary. Recent data from Eminli and colleagues found for example that progenitors and hematopoietic stem cells are far more amenable to reprogramming into iPSCs than are differentiated cells [3].

The optimization of the reprogramming technology is in full progress, the main goal being the generation of transgene free iPSCs. Different approaches are currently being tested, making use of transient factor delivery with vectors that do not integrate into the genome (adenoviruses, transfected plasmids, DNA minicircles) or through the use of expression-excision systems (Cre-LoxP) and lately by using transposons. A further approach towards alternatives to replace virally transduced transcription factors is the use of small chemical compounds to control the signaling cues responsible for reprogramming. Indeed, several reports described the possibility to reprogram fibroblasts to iPSCs through chemical complementation of some of the reprogramming factors [4,5].

Last but not least, besides the four Yamanaka factors, other factors such as LIN28 [6], Esrrb [7] and Nr5a2 [8] were also found to participate in reprogramming. The future will show how many - more or less potent - new genes are able to cooperate in the conversion of differentiated cells towards pluripotency. In this context, it is worth mentioning the recent discovery that it is possible to directly transdifferentiate mouse skin cells into functional murine nerve cells, without first generating iPSCs, and therefore directly inducing one cell type to become a completely different cell type by defined factors [9]. 

Our current knowledge of the genetic mechanisms regulating pluripotency and differentiation of ESCs and iPSCs are nevertheless not yet exhaustive. Some factors are since many years known to play an important role in maintenance of pluripotency, for example, LIF (leukaemia inhibiting factor) that activates the transcription factor STAT3, an essential component for the maintenance of the undifferentiated state of murine ES cells. Nevertheless, the genes targeted by STAT3 are not yet well characterized, and recent works showed that unknown genes acting downstream of this transcription factor are of great interest in this context [10]. The same holds true for other factors like Nanog, which is considered the key to pluripotency. However, its role is puzzling because it can be deleted from ESCs without causing them to differentiate, and it was astonishingly not present among the collection of genes that can induce reprogramming to iPSCs [11]. Nevertheless, Silva *et al.* could show that Nanog does help to separate incompletely reprogrammed cells from fully reprogrammed ones and is absolutely required for the acquisition of pluripotency. Cells that are not able to produce Nanog can still undergo the early stages of the reprogramming process, but cannot proceed to full pluripotency [12].

In a similar way, the controlled differentiation of pluripotent cells towards pure populations of precursors and terminally differentiated cells is also not yet completely understood. Although many of the conditions that facilitate lineage commitment and differentiation are known, a more precise knowledge of the genetic programs involved is a must before using stem cell therapies in humans, since undifferentiated stem cells transplanted in mice can cause tumor formation. 

The field of stem cell research is one of the grand challenges of our times and is fortunately strengthened through the advent of the modern high throughput technologies together with molecular biology, genetics, and pharmacology. The switch to the post-genomic era opens new possibilities in understanding the mechanism ruling cell fate. The integrative analysis of gene expression data, together with information on protein synthesis and modification will allow a much closer understanding of the cellular processes than the simple gene expression changes as measured today. 

The possibility to generate patient specific iPSCs allows, for example to step beyond analyzing generic genomes, and to understand which genetic differences between individuals are the keys for predispositions to certain diseases. 

The clarification of the correlations between pluripotency, epigenetics and DNA damage repair also need extensive studies. It is known that proliferating cells possess molecular mechanisms for sensing and repairing DNA lesions that occur at specifically DNA damage-activated cell cycle checkpoints. Unclear are the mechanisms present in progenitors of differentiated cells, in which cell cycle arrest is a critical signal to trigger the differentiation program, or in terminally differentiated cells, which are typically post-mitotic. How DNA lesions are detected, processed and repaired in these cells, remains an open question. Recent data indicated that cells undergoing reprogramming experience yet uncharacterized genotoxic stress [13], similar to that shown for oncogene activation in both *in vitro* and *in vivo* systems [14,15,16]. This observation highlights the existence of obstacles for the therapeutic potential of this approach and certainly calls for further molecular research to elucidate the close relationship between genome stability and cell reprogramming.

With this Special Issue of the journal Genes, we aim at presenting recent research and developments on this very exciting topic. Special interest is given to reports on genes and pathways involved in the establishment and maintenance of natural and acquired pluripotency, in controlling the global and local chromatin organization in pluripotent cells, and in triggering reprogramming in somatic and adult stem cells. We further aim at presenting reports on the mechanism controlling the switch from pluripotency to differentiation towards defined populations of cells. 
